# Thirty-Two Years of Public Health

**Published:** 1911-12-23

**Authors:** 


### THIRTY-TWO YEARS OF PUBLIC HEALTH.

### Interview with Dr. W. Collingridge on his Retirement.*

II.—THE HEALTH OF THE CITY.

### The Value of Women Inspectors.

Returning to his work in the City of London, Dr. Collingridge said: "There is one important difference between the work of the City medical officer and that of others. The City officer has no dealings with the population question. Our night population is so small."

"What is its ratio to the day population?"

"The figures are, roughly, 19,000 for our night population, while it is one and a half millions by day. We have the supervision of markets and factories in the City, of restaurants and dairies, and of the sale of food and drugs. For this work there are nine sanitary inspectors and ten food inspectors."

"Are any of these women?"

"Yes; that was one of my innovations. I found, as a little thought will make you realise, that women could get information on sanitary questions that men cannot at all easily do. Their duties comprise the inspection of the sanitary arrangements of workshops, factories, railway stations, and underground women's lavatories."

### Palatial Public Lavatories.

"The palatial attributes of our public lavatories date from about 1880, and the reason of their apparent luxury you may not suspect. They have been constructed so on the score of economy. Marble is cheap, of course, but the tiles, as in the latest example at the Royal Exchange lavatory, are lasting. The large area of them, their size, simply depends on the growth of the population."

"Reforms in them are connected with the City?"

"Yes; there is one of which I am particularly proud. We set the example of offering at least some free accommodation in the women's groups. It has now been followed up by several of the boroughs."

"How do the takings stand to expenses of upkeep?"

"Some pay their working expenses. But we must have public lavatories whether they pay or not, and their institution is the ideal example of municipal trading. There was no competition with private enterprise when they were started by the Corporation. They are necessary, and private enterprise had never tried, and could not run them at a profit. It was the place of the Corporation to step in. But as regards the introduction of factory inspection the Government must take credit for that. What we have done, however, is to introduce kitchens into workshops. That is a genuine improvement."

### The Truth about Restaurant Kitchens.

"Is there anything in the recurrent allegation that the kitchens of our restaurants would fill the public with horror if it only knew?"

"There has been an enormous improvement in the last ten years. There is none, I should say, which shows the evil conditions which prevailed ten years ago. One could find faults now no doubt, but they would concern mainly minor details."

"It would take me too long to cover the whole field of the inspection of tuberculous milk, of our researches into the relation of shell-fish to enteric; but there is the, perhaps, less realised inspection of hairdressers' shops."

### Hairdressers' Shops.

"The object was to prevent the spread of skin diseases. We have started a system of voluntary registration, which I hope one day will be enforced."

"How does the hairdresser get his *quid pro quo*?"

"In return for not using the shaving-brush for more than one customer without using an antiseptic—and as we have abolished hard soaps for antiseptic purposes the risk of barbers' itch is largely done away. The hair-brushes are washed daily, and though the transmission of nits is possible, it rarely occurs. This is not a very pleasant subject, though an important one—and as I say, in return for these precautions the barber is entitled to exhibit a certificate, a great document with the Corporation's seal and signed by the Town Clerk. His name also occurs in the annual report of the City Medical Officer. This advertisement has proved strong and good enough to make voluntary registration popular."

### Future Reforms in Meat Markets.

"As regards the Meat Markets?"

"There, as I think, things still need to be done. I should like to see the inspection of meat, at the time it is slaughtered, compulsory. That is the only time inspection is worth much, for it is only then that all the evidence is there. Then, too, all such inspected meat should be marked—not for the public's direct protection—they will never bother about it—but for the protection of the retail trader. In seven small private slaughter-houses in the City this is now being done. At present our practice is inconsistent. The public has a guarantee now for foreign meat, which is all marked. We want a guarantee for our own meat. The Food and Drugs Act, which gives us plenty to do, should cover this. As a proof of our activity, let me say that in 1901 there was in the number of milk samples analysed 21.2 per cent. of adulterated specimens. In 1910 the number was only 4.2. As to drugs, no fewer than 1,130 samples were analysed and showed 2.8 adulterated specimens. That shows the increase in the work in my time."

### On the Eve of Retirement.

"You are retiring before full term?'

"Yes; the work is now too much for me to cope with in the fulness that it demands from me."

"Have you any plans for the future?"

"Yes; I am not going to vegetate. I have had plenty of fighting in my time, and believe in it, so I have begun already to read for the Bar, and the rest of my time will be spent largely out of doors at my country home. I believe, as I told you, in a busy existence."

I did not like to press Dr. Collingridge further in the matter, but thought that his energy in reading for the Bar was worth recording, for it led him to make an interesting observation on the value of illustrations to books.

"In reading for law," he said, "I feel in Coke upon Littleton and the rest of that literature the absence of illustrations. My old medical studies and their textbooks were always illustrated, and medical illustration has indeed become an art of its own. I miss illustrations very much in my legal studies."

With this personal attitude our readers will probably agree, and we feel that they appreciated the excellent photograph that Dr. Collingridge's courtesy enabled us to publish as the best comment upon his conversation.

* The first part of this interview appeared in The Hospital of December 16.

